# Chitosan-MgO Nanocomposite: One Pot Preparation and Its Utility as an Ecofriendly Biocatalyst in the Synthesis of Thiazoles and [1,3,4]thiadiazoles

**DOI:** 10.3390/nano8110928

**Published:** 2018-11-08

**Authors:** Sayed M. Riyadh, Khaled D. Khalil, Ateyatallah Aljuhani

**Affiliations:** 1 Department of Chemistry, Faculty of Science, Taibah University, Al-Madinah Al-Mounawrah 30002, Saudi Arabia; riyadh1993@hotmail.com (S.M.R.); ateyatallah@hotmail.com (A.A.); 2 Department of Chemistry, Faculty of Science, Cairo University, Giza 12613, Egypt; 3 Department of Chemistry, Faculty of Science, Taibah University, Yanbu 46423, Saudi Arabia

**Keywords:** chitosan-MgO nanocomposite, heterogeneous catalysis, ethylidenethiosemicarbazides, thiazoles, thiadiazoles

## Abstract

A chitosan-MgO hybrid nanocomposite was prepared using a simple chemical precipitation method and characterized using Fourier transform spectroscopy (FTIR), elemental analysis (EDX), and scanning electron microscopy (SEM). The nanocomposite was served as a powerful ecofriendly basic catalyst under microwave irradiation in the synthesis of two novel series of 5-arylazo-2-hydrazonothiazoles **4a**–**j** and 2-hydrazono[1,3,4]thiadiazoles **8a**–**d**, incorporating a sulfonamide group. The structures of the synthesized products were elucidated by spectral data and elemental analyses. Also, their yield percentages were calculated using triethylamine (as a traditional catalyst) and chitosan-MgO nanocomposite (as a green recyclable catalyst) in a comparative study.

## 1. Introduction

**Figure 1:**
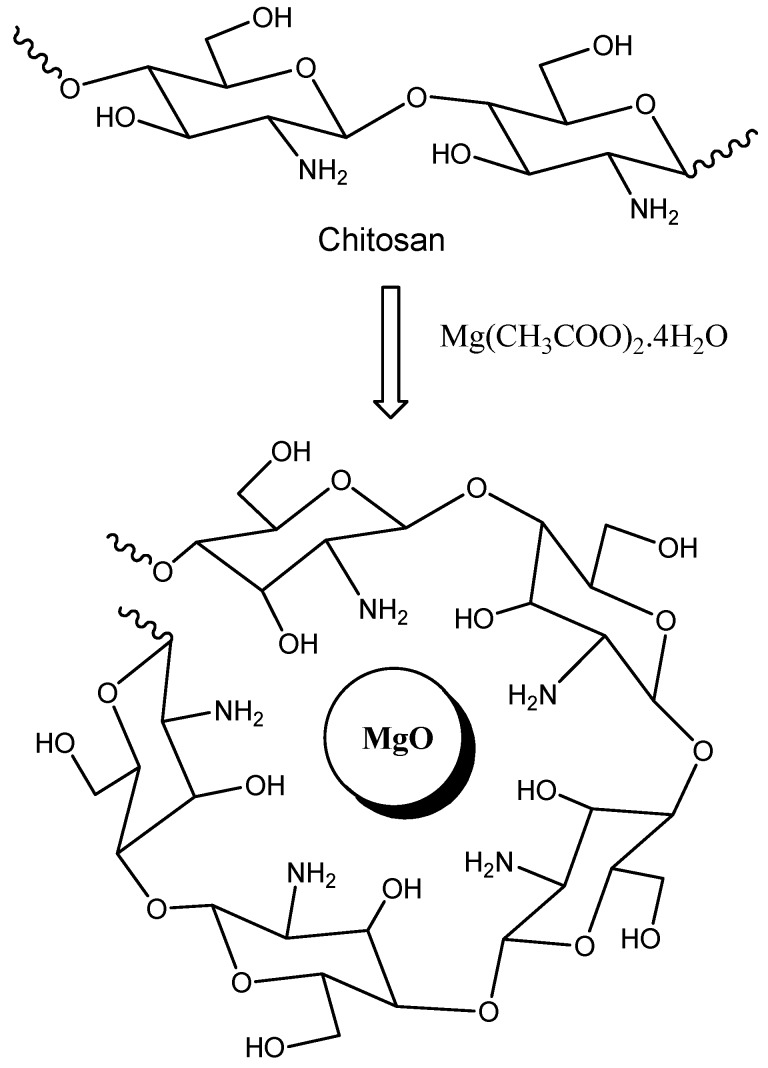
Structure of a chitosan-MgO nanocomposite.

In the past decade, green chemistry has been developed from a variety of research ideas such as atom economy and heterogeneous catalysis in the last few decades leading up to the 1990s, and in several successful attempts and efforts to overcome the problems of chemical pollution and resource depletion. On the other hand, nanosized materials are of great importance due to their large exposed surface area, high absorbability, and high catalytic efficiency. Nanosized magnesium oxides were being multitalented basic catalysts for many organic reactions. Recently, MgO nanoparticles can be used for the synthesis of pyranopyrazoles [1], aminochromenes [2], and dihydropyridines [3] via multicomponent reactions. The main drawbacks to the potential use of MgO nanoparticles as a basic catalyst are their difficult separation and reusability since the utilized catalyst could not recover quantitatively and the pure products should be obtained after extensive purification processes. Chitosan (CS) is a natural polysaccharide that is commercially produced via the alkaline hydrolysis of chitin. Although this biopolymer has many advantages, such as its renewability, biocompatibility, and biodegradability, its utility is limited in its unmodified form. For a long time, chitosan was used as an ecofriendly basic catalyst in some organic reactions [4,5,6,7]. However, the major problem of its use is the limited basic properties (weak catalytic activity) and its high swelling properties and gel formation renders its separation and recovery very difficult. To overcome these drawbacks, a chitosan-MgO nanocomposite [8] could be used as a novel basic heterogeneous catalyst in the form of a solid film. A chitosan-MgO nanocomposite is a three-dimensional, cross-linked, polymeric matrix of chitosan (with active NH and OH functional groups) incorporating magnesium oxide nanoparticles (Figure 1) [9].

The biocatalyst is stable enough such that it can be recovered and used more than four times without a loss in its catalytic activity. As a result of these studies, the chitosan-MgO biocatalyst was found to be ecofriendly, biocompatible, an efficient green catalyst, and a high economic impact material that will find a lot of potential catalytic applications, especially in the base catalyzed organic transformations. This heterogeneous hybrid nanocomposite is used in the form of films to increase its catalytic activity by increasing its surface/volume ratio, and consequently it can be easily recycled and reused several times with the same catalytic efficiency [10].

Moreover, thiazoles display a diversity of therapeutic activities such as being a non-steroidal anti-inflammatory drug (meloxicam), antiretroviral drug (ritonavir), antiprotozoal agent (nitazoxanide) [11], and antioxidant agent [12]. Also, [1,3,4]thiadiazoles with a sulfonamide group were incorporated in many marketing drugs including acetazolamide and methazolamide [13]. In this study, we are reporting an efficient green protocol and simple synthetic routes of a new series of hydrazonothiazoles and hydrazono [1,3,4]thiadiazoles in the presence of chitosan-MgO nanocomposite as a basic ecofriendly biocatalyst under microwave irradiations. Moreover, the efficiency of this biocatalyst and its reusability was examined.

## 2. Materials and Methods

Melting points were measured on an electrothermal Gallenkamp capillary apparatus (Leicester, UK) and are uncorrected. Elemental analyses were carried out at the Microanalytical Center of Cairo University, Giza, Egypt. The IR spectra were recorded on a Pye-Unicam SP300 Instrument (Cambridge, UK) in potassium bromide discs. The ^1^H NMR (nuclear magnetic resonance) and ^13^C NMR of the newly synthesized compounds in DMSO-*d*_6_ were measured on a Varian Mercury VXR-300 spectrometer (Varian, Karlsruhe, Germany) at 300 MHz for ^1^H NMR and 75 MHz for ^13^C NMR) and the chemical shifts were related to that of the solvent. The mass spectra were recorded on a GCMSQ1000-EX Shimadzu (Tokyo, Japan) and GCMS 5988-A HP spectrometers (Shimadzu, Tokyo, Japan) where the ionizing voltage was 70 eV. Microwave experiments were carried out using CEM Discover Labmate microwave apparatus (Discover, SP, NC, USA, 300 W). The starting materials 2-{1-[4-((4-methylphenyl)sulfonamide) phenyl]ethylidine}thiosemicarbazide **1** [14], *α*-keto hydrazonoyl halides **2a**–**j** [15,16,17,18], and arenecarbohydrazonoyl halides **5a**–**d** [19,20] were prepared as reported in literature.

### 2.1. Preparation of Heterogeneous Catalyst (Chitosan-MgO Hybrid Nanocomposite)

The hybrid CS-MgO nanocomposite was prepared using a one pot solution casting method. In an experiment, 2 g of chitosan powder (medium molecular weight; 85% DDA) was dissolved in 100 mL of 2% (*v*/*v*) aqueous acetic acid solution under stirring for 12 h at room temperature. To this solution, 2 g of magnesium acetate tetrahydrate, Mg(CH_3_COO)_2_·4H_2_O (M0631 Sigma-Aldrich, St. Louis, MO, USA) were added and the mixture was again re-stirred for 12 h till a clear solution was obtained. The resulting viscous solution was cast in a Teflon Petri dish and dried overnight at room temperature. The Petri dish was then immersed into a 0.2 M sodium hydroxide solution for neutralization and phase separation. The solid films were dried in an oven adjusted at 80 °C for 6 h. Finally, the produced solid films were purified by washing with methanol several times and again were dried in an oven at 60 °C for 2 h. Furthermore, a pure chitosan film, without MgO, was prepared in a similar way for comparative study.

### 2.2. Reactions of 2-{1-[4-((4-Methylphenyl)sulfonamide)phenyl]ethylidine}thiosemicarbazide (1) with α-Keto Hydrazonoyl Chlorides ***2a**–**j*** or N-Aryl Arenecarbohydrazonoyl Halides ***5a**–**d***

#### 2.2.1. Method A

A mixture of 2-{1-[4-((4-methylphenyl)sulfonamide)phenyl]ethylidine}thiosemicarbazide (1) (0.362 g, 1 mmol) in dry dioxane (15 mL), containing 0.1 g of triethylamine, and *α*-keto hydrazonoyl chlorides **2a**–**j** or *N*-aryl arenecarbohydrazonoyl halides **5a**–**d** (1 mmol of each) was irradiated using microwave irradiation (MW) at 300 W in a closed Teflon vessel until all the starting material was consumed (30–40 min as monitored by thin layer chromatography, TLC). The solvent was evaporated and the residue was poured into an ice/HCl mixture. The precipitate was filtered, washed with water, and crystallized from methanol to give products **4a**–**j** or **8a**–**d**.

#### 2.2.2. Method B

The procedure was similar to Method A, using a chitosan-MgO nanocomposite (0.1 g) instead of trimethylamine. After completion of the reaction, the hot solution was filtered to remove chitosan-MgO nanocomposite and the filtrate was poured into an ice/HCl mixture, and the precipitate was filtered, washed with water, and crystallized from methanol to give products **4a**–**j** or **8a**–**d** (see Supplementary Materials for analyses of the prepared compounds).

## 3. Results and Discussion

### 3.1. Preparation and Characterization of Chitosan-MgO Nanocomposite Films

The chitosan-MgO nanocatalyst was prepared via incorporation of the MgO nanoparticles in the chitosan matrix through a modified one pot solution casting method. The chitosan solution in acetic acid was treated with magnesium acetate tetrahydrate, then was subjected to evaporation of the solvent at room temperature. The resulting solid film was soaked in sodium hydroxide solution to achieve the phase separation.

#### 3.1.1. FTIR Spectra

**Figure 2:**
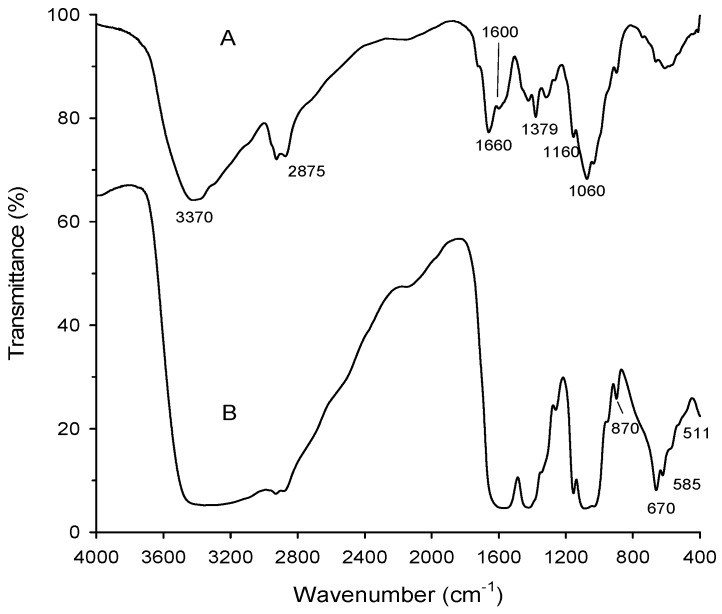
FTIR of chitosan (**A**) and the chitosan-MgO nanocomposite (**B**).

FTIR (Fourier-Transform infrared) spectra of chitosan and chitosan-MgO nanocomposite showed that the main functional groups of chitosan clearly appeared at *υ* = 3370 cm^−1^ (broad band of hydrogen bonded OH– groups), 2875 cm^−1^ (C–H bond; CH_3_ groups), 1660 cm^−1^ (amide carbonyl groups), 1379 cm^−1^ (bending vibration of CH_2_ groups), and 1060 cm^−1^ (asym. vibration of C=O) (Figure 2). These bands are considered as evidence for the maintenance of the chitosan structure features even after the incorporation of the MgO nanoparticles inside the polymer matrix. Also, the absence of acetic acid bands in the spectrum indicated that the films were washed enough and neutralized completely upon sodium hydroxide treatment (see Section 2.1). Moreover, only small acceptable shift in the bands of chitosan-MgO nanocomposite was attributed to the influence of the incorporation of MgO nanoparticles. This shift in bands was familiar as result of chitosan with metal oxides [21]. The latter shifts are shown, especially at bands of NH and OH groups, which is evidence for the H-bonding interaction of these group with MgO molecules.

#### 3.1.2. FESEM Analysis

**Figure 3:**
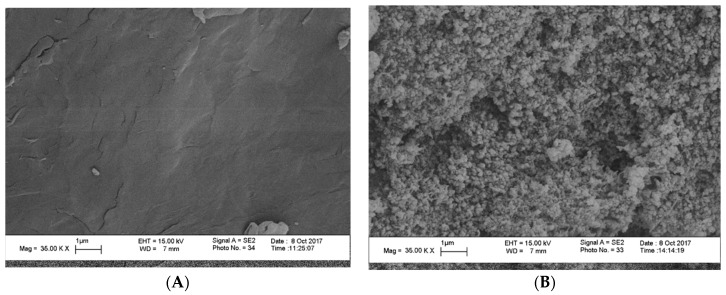
FESEM images of chitosan (**A**), and chitosan-MgO 10 wt% (**B**).

**Figure 4:**
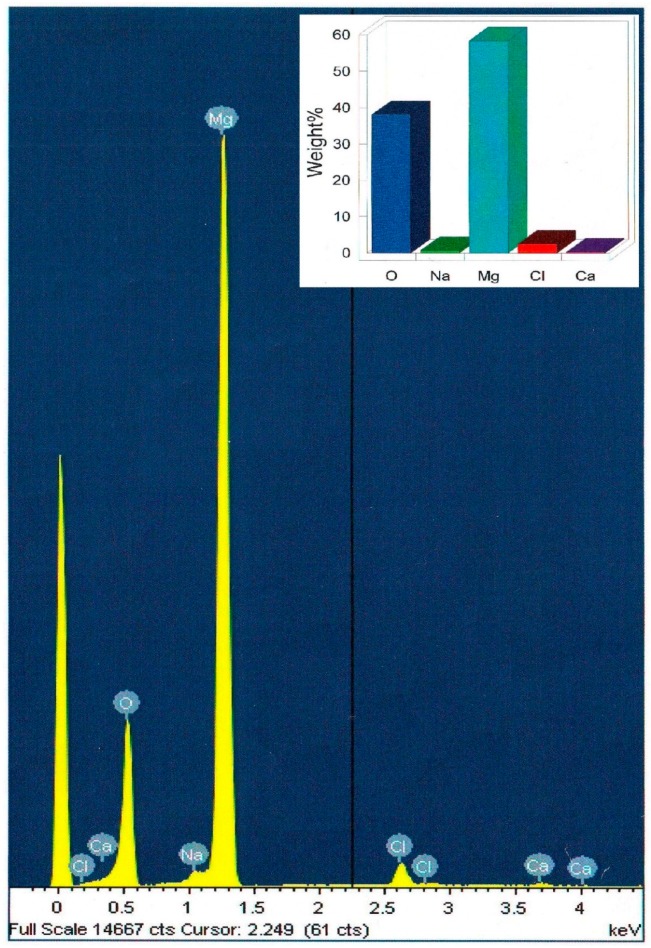
EDX spectra of the 10 wt% chitosan-MgO nanocomposite hybrid film. Inset is the quantitative result.

FESEM (Field Emission Scanning Electron Microscope) was used to analyze the morphology and size distribution of the MgO nanoparticles that were incorporated in the polymer matrix. FESEM micrographs of the pure chitosan (A) and that of the hybrid films with magnesia particles 10 wt% (B) are shown in (Figure 3). The obtained surface of the pure chitosan matrix was found to be homogenous and looks smoothly to a great extent. The MgO nanoparticles developed as white spots that distributed homogeneously over the surface of the polymer matrix. On the other hand, the muddled surface was due to adsorption of the polymer layers on the particles surface. The average size of the MgO particles was found to be approximately 6–11 nm for 10 wt% as the particles size slightly decreased with increasing magnesia content. Moreover, EDX measurements for the solid chitosan-MgO nanocomposite confirmed the presence of magnesia within the hybrid film, as shown in (Figure 4).

### 3.2. Optimal Catalyst Loading

**Figure 5:**
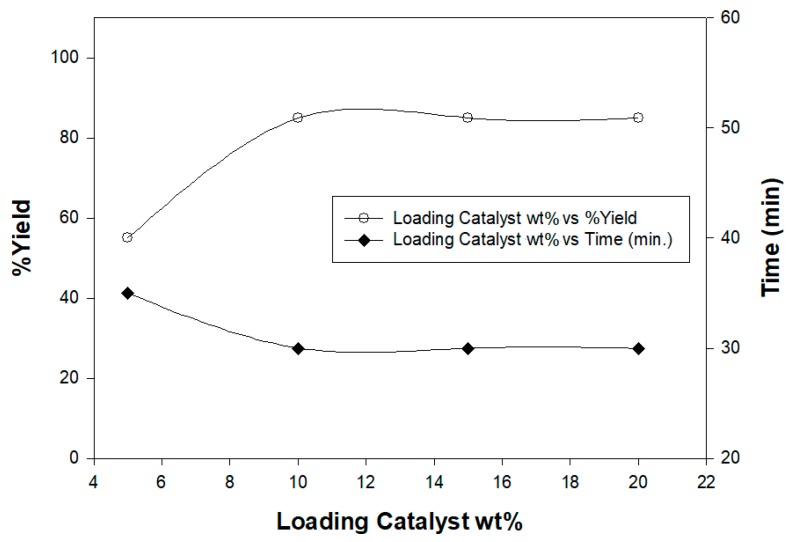
Optimization of the chitosan graft copolymer as a basic catalyst.

In order to estimate the proper catalyst loading, a model irradiation reaction of ethylidinethiosemicarbazide **1** with *α*-keto hydrazonoyl chlorides **2a** in dioxane, and in the presence of 1, 5, 10, 15, and 20 wt% of chitosan-MgO nanocomposites as basic catalysts, under the same conditions was conducted affording the respective hydrazonothiazoles **4a** (Figure 5). The results showed that the optimal catalyst loading was 10 wt% Moreover, the catalyst was reused four time and the results also showed that the biocatalyst could be reused as such without significant loss in its catalytic activity (Table 1).

### 3.3. Synthesis of Thiazoles and [1,3,4]thiadiazoles Using Cs-MgO Nanocomposite

**Scheme 1:**
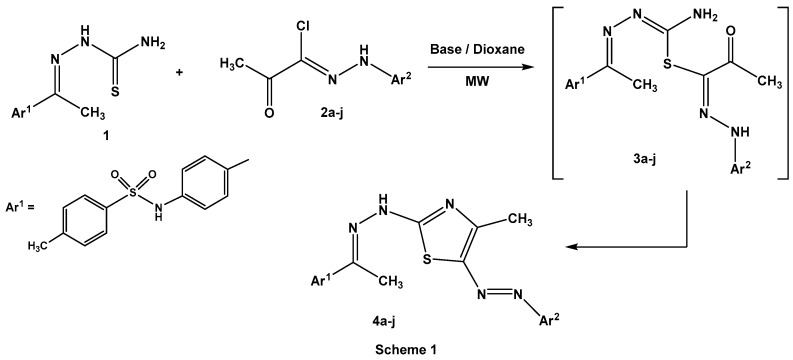
Synthesis of hydrazonothiazoles **4a**–**j**.

Irradiation of thiosemicarbazone **1** with 2-oxo-*N*-arylpropanehydrazonoyl chlorides **2a**–**j** in dioxane, in the presence of triethylamine or the chitosan-MgO nanocomposite as a basic catalyst, furnished 4-methyl-5-arylazo-2-hydrazonothiazoles **4a**–**j** (Scheme 1). At the outset, identification of the best basic catalyst (triethylamine or chitosan-MgO nanocomposite) was examined under microwave irradiations (Table 2).

As shown in Table 2, the reaction proceeded smoothly with different substituents on the aromatic benzene ring of hydrazonoyl chlorides **2a**–**j**. Also, the chitosan-MgO nanocomposite was a more efficient basic catalyst than triethylamine under microwave irradiation.

Elucidation of 2-hydrazonothiazoles **4a**–**j** structures was based on spectral data and elemental analyses. In the IR spectra, two absorption bands in the range of *υ* = 3212–3268 cm^−1^ and 1578–1600 cm^−1^ were revealed owing to the presence of (N–H) and (C=N) groups, respectively. Also, the sulfonamide group (SO_2_NH) showed asymmetric and symmetric stretching signals at *υ* = 1336–1379 cm^−1^ and 1136–1161 cm^−1^, respectively [22]. In ^1^H NMR spectra two methyl groups bordering to hydrazone moiety (CH_3_–C=N–NH) [23] and a thiazole ring [24] were observed as singlet signals at *δ* = 2.35–2.43 and 2.43–2.59 ppm, respectively, while the NH proton of the sulfonamide group [22] was resonated at *δ* = 10.43–10.92 ppm.

**Scheme 2:**
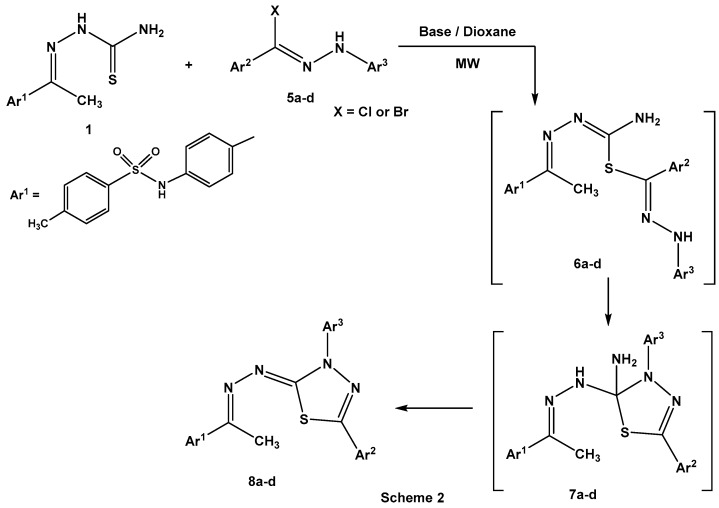
Synthesis of hydrazono[1,3,4]thiadiazoles **8a**–**d**.

Establishing the experimental feasibility of the reaction of **1** with *α*-keto hydrazonoyl chlorides **2a**–**j** directed our attention to use *N*-aryl arenecarbohydrazonoyl halides **5a**–**d**, bereft of the carbonyl group. Thus, treatment of ethylidinethiosemicarbazide **1** with *N*-aryl arenecarbohydrazonoyl halides **5a**–**d** under the same employed conditions proceeded smoothly to give 2-hydrazono[1,3,4] thiadiazoles **8a**–**d** as the isolated products (Scheme 2). Also, the effect of the nature of the basic catalyst, such as triethylamine or the chitosan-MgO nanocomposite, on the percent yields of the isolated products **8a**–**d** was investigated (Table 3).

As shown in Table 3, the percentage yields of the products **8a**–**d**, the chitosan-MgO nanocomposite as a basic catalyst prevailed over triethylamine under microwave irradiation. The characterization of 2-hydrazono[1,3,4]thiadiazoles **8a**–**d** was consistent with spectral data (IR, ^1^H NMR, ^13^C NMR, and MS (Mass spectroscopy)) and elemental analyses (see Supplementary Materials). As shown in Scheme 2, the reaction proceeded through nucleophilic displacement of the thiol group to the halogen atom to give *S*-alkylated intermediate products **6a**–**d** [25]. Intramolecular Michael addition [26] of the NH group into the electrophilic carbon atom of (C=N-N=) for intermediates **6a**–**d** led to the formation of cycloadducts **7a**–**d**. Elimination of ammonia from the latter intermediates **7a**–**d** gave the final products **8a**–**d** (Scheme 2).

## 4. Conclusions

Recently, nanoparticles (NPs) have been developed as promising candidates in various applications due to their unique properties. In this article, a chitosan-MgO nanocomposite (as a green recyclable biocatalyst) was prepared and well-characterized using FTIR, FESEM, and EDX spectra. The average size of the MgO particles was found to be approximately 6–11 nm for 10 wt% and it was found that the particles’ size slightly decreased with increasing magnesia content. This nanocomposite was then used successfully as a heterogeneous basic catalyst for the synthesis of two series of hydrazonothiazoles and hydrazono [1,3,4] thiadiazoles, with sulfonamide moiety, in a comparative study with triethylamine (as a traditional catalyst). In addition to the preferable green impact, the acquired results showed that the chitosan-MgO nanocomposite was a more powerful catalyst in these reactions as compared to triethylamine. The obvious catalytic potency of the chitosan-MgO nanocomposite was attributed to the obtained nanosized MgO and the synergistic effect that is created by the combination of the basic nature of both MgO and chitosan. Moreover, the nanocatalyst could be easily recovered and reused many times without loss in its catalytic activity. Finally, the biopolymer chitosan-metal oxide combination is a promising hybrid nanocomposite that deserves to be explored in many organic transformations.

## Figures and Tables

**Table 1:** Recyclability of the chitosan graft copolymer as basic catalyst.

State of Catalyst	Fresh Catalyst	Recycled (1)	Recycled (2)	Recycled (3)	Recycled (4)
**Product 4a (%Yield)**	85	84	83	83	82

**Table 2:** Effect of the nature of basic catalyst on the yield of the products **4a**–**j**.

Compound Number	Ar^2^	Time (min)	Yield (%)	
			Et_3_N	Chitosan-MgO Nanocomposite
**4a**	C_6_H_5_	30	76	85
**4b**	2-CH_3_C_6_H_4_	30	73	85
**4c**	2-ClC_6_H_4_	35	77	86
**4d**	3-CH_3_C_6_H_4_	30	82	96
**4e**	4-CH_3_C_6_H_4_	30	78	87
**4f**	4-CH_3_OC_6_H_4_	40	73	83
**4g**	4-NO_2_C_6_H_4_	40	69	81
**4h**	4-BrC_6_H_4_	40	70	81
**4i**	4-FC_6_H_4_	40	71	80
**4j**	4-CH_3_COC_6_H_4_	40	72	84

**Table 3:** Effect of the nature of basic catalyst on the yield of the products **8a**–**d**.

Compound Number	Ar^2^	Ar^3^	Time (min)	Yield (%)	
				Et_3_N	Chitosan/MgO Nanocomposite
**8a**	C_6_H_5_	C_6_H_5_	30	74	86
**8b**	C_6_H_5_	4-NO_2_C_6_H_4_	35	76	88
**8c**	4-CH_3_C_6_H_4_	C_6_H_5_	40	79	85
**8d**	4-CH_3_OC_6_H_4_	C_6_H_5_	40	78	87

## Supplementary Materials

The following are available online at http://www.mdpi.com/2079-4991/8/11/928/s1.
