# Supplementary material for: Chitosan-MgO Nanocomposite: One Pot Preparation and Its Utility as an Ecofriendly Biocatalyst in the Synthesis of Thiazoles and [1,3,4]thiadiazoles
Source: Nanomaterials (Basel). 2018 Nov 8;8(11):928. doi: 10.3390/nano8110928 (PMC6266359; doi:10.3390/nano8110928)
Supplement: Supplementary file 1 [file nanomaterials-08-00928-s001.pdf]

Supplementary Data

Chitosan-MgO Nanocomposite: One Pot Preparation and Its Utility as an Ecofriendly Biocatalyst in the Synthesis of Thiazoles and [1,3,4]thiadiazoles

Sayed M. Riyadh [1,2], Khaled D. Khalil [2,3,*], and Ateyatallah Aljuhani [1]

[1] Department of Chemistry, Faculty of Science, Taibah University, Al-Madinah Al-Mounawrah 30002, Saudi Arabia; riyadh1993@hotmail.com (S.M.R.); ateyatallah@hotmail.com (A.A.)

[2] Department of Chemistry, Faculty of Science, Cairo University, Giza 12613, Egypt

[3] Department of Chemistry, Faculty of Science, Taibah University, Yanbu 46423, Saudi Arabia

* Correspondence: khd.khalil@yahoo.com

2-{2-[1-(4-(4-Methylphenyl)sulfonamide)ethylidene]hydrazono}-4-methyl-5-(phenylazo) thiazole (4a)

Red crystals; mp. 218–220 °C; IR (KBr) 3260 (NH), 1597 (C=N), 1378, 1157 (SO_2–N) cm^{-1}; ^{1}H-NMR (DMSO-d_6) = 2.29 (3H, s, Ar–CH_3), 2.35 (3H, s, CH_3–C=N–NH), 2.49 (3H, s, thiazole–CH_3), 6.95–7.80 (13H, m, Ar–H + 1H, s, NH), 10.43 (1H, s, SO_2–NH) ppm; ^{13}C-NMR (DMSO-d_6) = 14.90 (CH_3), 16.30 (CH_3), 20.92 (CH_3), 114.72, 118.67, 122.26, 126.75 (two merged signals), 127.95 (two merged signals), 129.25 (two merged signals), 129.80 (two merged signals), 132.48, 136.52, 138.09, 140.03, 143.62 ppm; MS *m/z* (%): 504 (M^+, 80), 400 (57), 349 (44), 287 (78), 91 (100), 77 (44). Anal. Calcd for $C_{25}H_{24}N_6O_2S_2$ (504.14): C, 59.50; H, 4.79; N, 16.65; S, 12.71. Found: C, 59.52; H, 4.81; N, 16.81; S, 12.94 %.

2-{2-[1-(4-(4-Methylphenyl)sulfonamide)ethylidene]hydrazono}-4-methyl-5-[(2-methyl-phenyl)azo]thiazole (4b)

Brown powder; mp. 216–218 °C; IR (KBr) 3212 (NH), 1599 (C=N), 1336, 1156 (SO_2–N) cm^{-1}; ^{1}H-NMR (DMSO-d_6) = 2.23 (3H, s, Ar–CH_3), 2.27 (3H, s, Ar–CH_3), 2.38 (3H, s, CH_3–C=N–NH), 2.55 (3H, s, thiazole–CH_3), 7.02–7.83 (12H, m, Ar–H), 10.62 (1H, s, C=N–NH), 10.87 (1H, s, SO_2–NH) ppm; ^{13}C-NMR (DMSO-d_6) = 14.95 (CH_3), 17.70 (CH_3), 21.06 (CH_3), 26.44 (CH_3), 116.48, 118.12, 119.02 (two merged signals), 126.76 (two merged signals), 126.87, 127.84, 129.86 (two merged signals), 129.92, 131.14, 132.75, 136.90, 140.02, 143.53, 143.73 ppm; MS *m/z* (%): 518 (M^+, 29), 490 (30), 287 (75), 91 (100). Anal. Calcd for $C_{26}H_{26}N_6O_2S_2$ (518.16): C, 60.21; H, 5.05; N, 16.20; S, 12.36. Found: C, 60.40; H, 4.85; N, 16.43; S, 12.28 %.

2-{2-[1-(4-(4-Methylphenyl)sulfonamide)ethylidene]hydrazono}-4-methyl-5-[(2-chloro-phenyl)azo]thiazole (4c)

Orange microcrystals; mp. 160–162 °C; IR (KBr) 3232 (NH), 1596 (C=N), 1375, 1158 (SO_2–N) cm^{-1}; ^{1}H-NMR (DMSO-d_6) = 2.30 (3H, s, Ar–CH_3), 2.36 (3H, s, CH_3–C=N–NH), 2.54 (3H, s, thiazole–CH_3), 7.16–7.78 (12H, m, Ar–H), 9.10 (1H, s, C=N–NH), 10.57 (1H, s, SO_2–NH) ppm; ^{13}C-NMR (DMSO-d_6) = 14.05 (CH_3), 14.98 (CH_3), 20.72 (CH_3), 111.73, 114.78, 115.49, 118.08, 118.89, 122.65, 123.16, 126.88, 128.08, 129.22, 129.60, 129.92, 132.68, 136.76, 138.76, 140.19, 143.55, 143.73 ppm; MS *m/z* (%): 540 (M^{+2}, 8), 538 (M^+, 25), 338 (30), 287 (50), 91 (100). Anal. Calcd for $C_{25}H_{23}ClN_6O_2S_2$ (539.10): C, 55.70; H, 4.30; N, 15.59; S, 11.89. Found: C, 55.45; H, 4.05; N, 15.34; S, 11.65 %.

2-{2-[1-(4-(4-Methylphenyl)sulfonamide)ethylidene]hydrazono}-4-methyl-5-[(3-methyl-phenyl)azo]thiazole (4d)

Brown powder; mp. 178–180 °C; IR (KBr) 3261 (NH), 1598 (C=N), 1375, 1158 (SO_2–N) cm^{-1}; ^{1}H-NMR (DMSO-d_6) = 2.26 (3H, s, Ar–CH_3), 2.30 (3H, s, Ar–CH_3), 2.36 (3H, s, CH_3–C=N–NH), 2.43 (3H, s, thiazole–CH_3), 6.85–7.81 (12H, m, Ar–H), 10.39 (1H, s, C=N–NH), 10.61 (1H, s, SO_2–NH) ppm; ^{13}C-

NMR (DMSO-d_6) = 15.05 (CH_3), 16.50 (CH_3), 21.05 (CH_3), 21.34 (CH_3), 111.73, 114.78, 115.49, 118.08, 118.89, 123.16, 126.88 (two merged signals), 128.08, 129.22, 129.60, 129.92, 132.68, 136.76, 138.76, 140.19, 143.55, 143.73 ppm; MS *m/z* (%): 518 (M^+, 53), 490 (40), 287 (57), 91 (100). Anal. Calcd for $C_{26}H_{26}N_6O_2S_2$ (518.16): C, 60.21; H, 5.05; N, 16.20; S, 12.36. Found: C, 60.44; H, 4.95; N, 16.39; S, 12.21 %.

2-{2-[1-(4-(4-Methylphenyl)sulfonamide)ethylidene]hydrazono}-4-methyl-5-[(4-methylphenyl)azo]thiazole (4e)

Red microcrystals; mp. 121–123 °C; IR (KBr) 3262 (NH), 1597 (C=N), 1374, 1155 (SO_2-N) cm^{-1}; ^{1}H-NMR (DMSO-d_6) = 2.26 (3H, s, Ar–CH_3), 2.31 (3H, s, Ar–CH_3), 2.40 (3H, s, CH_3–C=N–NH), 2.55 (3H, s, thiazole–CH_3), 7.12–7.83 (12H, m, Ar–H), 10.39 (1H, s, C=N-NH), 10.54 (1H, s, SO_2–NH) ppm; ^{13}C-NMR (DMSO-d_6) = 15.21 (CH_3), 16.72 (CH_3), 20.52 (CH_3), 21.08 (CH_3), 111.71, 114.65, 115.49, 118.11, 118.91, 123.14, 126.87, 128.15, 129.22, 129.57, 129.90, 132.45, 136.72, 138.74, 141.19, 143.51 ppm; MS *m/z* (%): 518 (M^+, 40), 490 (40), 287 (50), 91 (100). Anal. Calcd for $C_{26}H_{26}N_6O_2S_2$ (518.16) : C, 60.21; H, 5.05; N, 16.20; S, 12.36. Found: C, 60.42; H, 5.19; N, 16.11; S, 12.45 %.

2-{2-[1-(4-(4-Methylphenyl)sulfonamide)ethylidene]hydrazono}-4-methyl-5-[(4-methoxyphenyl)azo]thiazole (4f)

Black crystals; mp. 138–140 °C; IR (KBr) 3268 (NH), 1594 (C=N), 1371, 1154 (SO_2–N) cm^{-1}; ^{1}H-NMR (DMSO-d_6) = 2.29 (3H, s, Ar–CH_3), 2.43 (3H, s, CH_3–C=N–NH), 2.55 (3H, s, thiazole–CH_3), 3.05 (3H, s, Ar–OCH_3), 6.89–7.80 (12H, m, Ar–H), 10.55 (1H, s, C=N–NH), 10.79 (1H, s, SO_2–NH) ppm; ^{13}C-NMR (DMSO-d_6) = 15.87 (CH_3), 21.04 (CH_3), 26.44 (CH_3), 55.49 (OCH_3), 114.78, 117.34, 118.11, 119.01, 124.61, 126.88, 127.84, 129.85, 129.89, 129.95, 132.08, 132.81, 136.89, 139.95, 142.50, 143.68 ppm; MS *m/z* (%): 534 (M^+, 22), 506 (70), 490 (100), 287 (75), 91 (90). Anal. Calcd for $C_{26}H_{26}N_6O_3S_2$ (534.15): C, 58.41; H, 4.90; N, 15.72; S, 11.99. Found: C, 58.22; H, 5.03; N, 15.91; S, 12.12 %.

2-{2-[1-(4-(4-Methylphenyl)sulfonamide)ethylidene]hydrazono}-4-methyl-5-[(4-nitrophenyl)azo]thiazole (4g)

Red powder; mp. 260–262 °C; IR (KBr) 3259 (NH), 1594 (C=N), 1378, 1152 (SO_2–N) cm^{-1}; ^{1}H-NMR (DMSO-d_6) = 2.32 (3H, s, Ar–CH_3), 2.38 (3H, s, CH_3–C=N–NH), 2.55 (3H, s, thiazole–CH_3), 7.15–8.16 (12H, m, Ar–H), 10.54 (1H, s, C=N–NH), 10.92 (1H, s, SO_2–NH) ppm; ^{13}C-NMR (DMSO-d_6) = 15.20 (CH_3), 16.59 (CH_3), 21.05 (CH_3), 113.93, 118.80, 120.61, 120.64, 125.78, 126.35, 126.86, 127.84, 128.23, 129.11, 129.94, 132.34, 132.81, 136.89, 140.46, 143.62 ppm; MS *m/z* (%): 549 (M^+, 13), 516 (30), 288 (30), 91 (100). Anal. Calcd for $C_{25}H_{23}N_7O_4S_2$ (549.13): C, 54.63; H, 4.22; N, 17.84; S, 11.67. Found: C, 54.50; H, 4.15; N, 17.60; S, 11.73 %.

2-{2-[1-(4-(4-Methylphenyl)sulfonamide)ethylidene]hydrazono}-4-methyl-5-[(4-bromophenyl)azo]thiazole (4h)

Orange microcrystals; mp. 243–245 °C; IR (KBr) 3262 (NH), 1598 (C=N), 1379, 1159 (SO_2–N) cm^{-1}; ^{1}H-NMR (DMSO-d_6) = 2.31 (3H, s, Ar–CH_3), 2.37 (3H, s, CH_3–C=N–NH), 2.51 (3H, s, thiazole–CH_3), 7.14–7.81 (12H, m, Ar–H), 10.50 (1H, s, C=N–NH), 10.58 (1H, s, SO_2–NH) ppm; ^{13}C-NMR (DMSO-d_6) = 14.97 (CH_3), 16.59 CH_3), 20.94 (CH_3), 116.14, 118.66, 120.62, 121.64, 125.78, 126.75, 126.96, 127.84, 128.00, 129.82, 129.94, 131.98, 132.39, 136.50, 140.10, 143.65 ppm; MS *m/z* (%):584 (M^{+2}, 44), 582 (M^+, 41), 429 (13), 287 (56), 91 (100). Anal. Calcd for $C_{25}H_{23}BrN_6O_2S_2$ (582.05): C, 51.46; H, 3.97; N, 14.40; S, 10.99. Found: C, 51.70; H, 4.12; N, 14.62; S, 11.12 %.

2-{2-[1-(4-(4-Methylphenyl)sulfonamide)ethylidene]hydrazono}-4-methyl-5-[(4-fluorophenyl)azo]thiazole (4i)

Brown powder; mp. 208–210 °C; IR (KBr) 3260 (NH), 1600 (C=N), 1372, 1156 (SO_2–N) cm^{-1}; ^{1}H-NMR (DMSO-d_6) = 2.32 (3H, s, Ar–CH_3), 2.39 (3H, s, CH_3–C=N–NH), 2.54 (3H, s, thiazole–CH_3), 7.13–7.83 (12H, m, Ar–H), 10.62 (1H, s, C=N–NH), 10.83 (1H, s, SO_2–NH) ppm; ^{13}C-NMR (DMSO-d_6) = 15.06 (CH_3), 16.39 (CH_3), 21.09 (CH_3), 115.90, 116.20, 118.02, 118.89, 120.48, 121.63, 126.75, 126.89, 127.84,

128.02, 129.91, 131.98, 132.56, 136.77, 140.25, 143.62 ppm; MS *m/z* (%): 522 (M^+, 40), 287 (60), 132 (30), 91 (100). Anal. Calcd for $C_{25}H_{23}FN_6O_2S_2$ (522.13): C, 57.46; H, 4.44; N, 16.08; S, 12.27. Found: C, 57.70; H, 4.22; N, 16.23; S, 12.12 %.

2-{2-[1-(4-(4-Methylphenyl)sulfonamide)ethylidene]hydrazono}-4-methyl-5-[(4-acetyl-phenyl)azo]thiazole (4j)

Brown powder; mp. 198–200 °C; IR (KBr) 3218 (NH), 1599 (C=N), 1375, 1161 (SO_2–N) cm^{-1}; ^{1}H-NMR (DMSO-d_6) = 2.31 (3H, s, Ar–CH_3), 2.40 (3H, s, CH_3–C=N–NH), 2.59 (3H, s, thiazole–CH_3), 3.33 (3H, s, Ar–$COCH_3$), 7.16–7.99 (12H, m, Ar–H), 10.54 (1H, s, C=N–NH), 10.74 (1H, s, SO_2–NH) ppm; ^{13}C-NMR (DMSO-d_6) = 15.04 (CH_3), 20.93 (CH_3), 26.32 (CH_3), 30.93 ($COCH_3$), 113.63, 114.42, 117.96, 118.73, 119.11, 124.65, 126.75, 128.08, 129.75, 129.86, 130.15, 132.84, 136.59, 139.93, 142.50, 143.74, 171.65 (C=O) ppm; MS *m/z* (%): 546 (M^+, 15), 391 (15), 288 (40), 91 (100). Anal. Calcd for $C_{27}H_{26}N_6O_3S_2$ (546.15): C, 59.32; H, 4.79; N, 15.37; S, 11.73. Found: C, 59.22; H, 4.93; N, 15.11; S, 11.62 %.

2-{2-[1-(4-(4-Methylphenyl)sulfonamide)ethylidene]hydrazono}-3,5-diphenyl-2,3-dihydro [1,3,4]thiadiazole (8a)

Yellow crystals; mp. 180–182 °C; IR (KBr) 3325 (NH), 1600 (C=N), 1375, 1155 (SO_2–N) cm^{-1}; ^{1}H-NMR (DMSO-d_6) = 2.33 (3H, s, Ar–CH_3), 2.37 (3H, s, CH_3–C=N–N=), 7.15–8.16 (18H, m, Ar–H), 10.50 (1H, s, SO_2–NH) ppm; ^{13}C-NMR (DMSO-d_6) = 15.62 (CH_3), 21.44 (CH_3), 119.41, 121.53, 123.45, 125.18, 126.41, 126.69, 127.20, 127.82, 129.48, 129.79, 130.26, 131.59, 133.19, 137.00, 139.96, 143.87, 150.99, 158.69, 163.94 ppm; MS *m/z* (%): 539 (M^+, 100), 378 (21), 77 (45). Anal. Calcd for $C_{29}H_{25}N_5O_2S_2$ (539.14): C, 64.54; H, 4.67; N, 12.98; S, 11.88. Found: C, 64.68; H, 4.79; N, 13.15; S, 12.10 %.

2-{2-[1-(4-(4-Methylphenyl)sulfonamide)ethylidene]hydrazono}-3-(4-nitrophenyl)-5-phenyl-2,3-dihydro[1,3,4]thiadiazole (8b)

Pale orange crystals; mp. 202–204 °C; IR (KBr) 3300 (NH), 1599 (C=N), 1372, 1155 (SO_2–N) cm^{-1}; ^{1}H-NMR (DMSO-d_6) = 2.33 (3H, s, Ar–CH_3), 2.40 (3H, s, CH_3–C=N–N=), 7.16–8.50 (17H, m, Ar–H), 10.56 (1H, s, SO_2–NH) ppm; ^{13}C-NMR (DMSO-d_6) = 15.89 (CH_3), 21.39 (CH_3), 119.21, 120.59, 125.27, 126.91, 127.11, 128.03, 129.48, 129.72, 130.24, 132.11, 132.81, 137.03, 139.96, 143.91, 144.85, 152.29, 152.59, 160.43, 163.66 ppm; MS *m/z* (%): 584 (M^+, 71), 568 (31), 378 (100), 77 (45). Anal. Calcd for $C_{29}H_{24}N_6O_4S_2$ (584.13): C, 59.58; H, 4.14; N, 14.37; S, 10.97. Found: C, 59.68; H, 4.28; N, 14.54; S, 11.10 %.

2-{2-[1-(4-(4-Methylphenyl)sulfonamide)ethylidene]hydrazono}-3-phenyl-5-(4-methoxy-phenyl)-2,3-dihydro[1,3,4]thiadiazole (8c)

Yellow crystals; mp. 192–194 °C; IR (KBr) 3225 (NH), 1600 (C=N), 1370, 1157 (SO_2–N) cm^{-1}; ^{1}H-NMR (DMSO-d_6) = 2.33 (3H, s, Ar–CH_3), 2.37 (3H, s, CH_3–C=N–N=), 3.84 (3H, s, OCH_3), 7.06–8.16 (17H, m, Ar–H), 10.50 (1H, s, SO_2–NH) ppm; ^{13}C-NMR (DMSO-d_6) = 15.56 (CH_3), 21.39 (CH_3), 55.94 (OCH_3), 115.12, 119.37, 120.99, 121.35, 122.54, 126.13, 127.16, 127.75, 128.33, 129.40, 130.23, 133.31, 137.03, 139.47, 139.99, 143.88, 150.74, 161.80, 164.26 ppm; MS *m/z* (%): 569 (M^+, 100), 415 (49), 378 (70), 77 (45). Anal. Calcd for $C_{30}H_{27}N_5O_3S_2$ (569.16): C, 63.25; H, 4.78; N, 12.29; S, 11.26. Found: C, 63.09; H, 4.53; N, 12.04; S, 11.15 %.

2-{2-[1-(4-(4-Methylphenyl)sulfonamide)ethylidene]hydrazono}-3-phenyl-5-(4-methyl-phenyl)-2,3-dihydro[1,3,4]thiadiazole (8d)

Yellowish green crystals; mp. 218–220 °C; IR (KBr) 3280 (NH), 1600 (C=N), 1373, 1155 (SO_2–N) cm^{-1}; ^{1}H-NMR (DMSO-d_6) = 2.22 (3H, s, Ar–CH_3), 2.34 (3H, s, Ar–CH_3), 2.41 (3H, s, CH_3–C=N–N=), 7.06–8.09 (17H, m, Ar–H), 10.52 (1H, s, SO_2–NH) ppm; ^{13}C-NMR (DMSO-d_6) = 15.58 (CH_3), 16.31 (CH_3), 21.39 (CH_3), 115.77, 116.45, 120.94, 121.12, 122.11, 125.93, 126.86, 127.73, 128.21, 129.43, 130.77, 132.91, 136.85, 139.27, 140.05, 143.85, 150.72, 161.94, 164.14 ppm; MS *m/z* (%): 553 (M^+, 28), 470 (100), 378 (55), 77 (45). Anal. Calcd for $C_{30}H_{27}N_5O_2S_2$ (553.16): C, 65.08; H, 4.92; N, 12.65; S, 11.58. Found: C, 64.99; H, 4.73; N, 12.44; S, 11.42 %.
